# Effects on the Caco-2 Cells of a Hypoglycemic Protein from Lupin Seeds in a Solution and Adsorbed on Polystyrene Nanoparticles to Mimic a Complex Food Matrix

**DOI:** 10.3390/biom9100606

**Published:** 2019-10-14

**Authors:** Alberto Barbiroli, Jessica Capraro, Serena Marulo, Marta Gamba, Alessio Scarafoni

**Affiliations:** 1 Department of Food, Environmental and Nutritional Sciences, Università degli Studi di Milano, 20133 Milano, Italy; alberto.barbiroli@unimi.it (A.B.); jessica.capraro@unimi.it (J.C.); marta.gamba@studenti.unimi.it (M.G.); 2 Department of Agricultural Sciences, Università degli Studi di Napoli Federico II, 80055 Portici (Napoli), Italy; serena.marulo@gmail.com

**Keywords:** γ-conglutin, seed proteins, bioactive proteins, structure/function relationships, *Lupinus albus*

## Abstract

The search for bioactivities influencing the human wellbeing of food proteins and peptides is a topic of broad and current interest. γ-Conglutin (γC) is a lupin seed protein drawing remarkable pharmacological and/or nutraceutical interest, as it is able to reduce hyperglycemia in humans and animal models. The present work deepens our investigations to understand the molecular basis of the in vitro effects of γC by testing the possible metabolic effects on cultivated Caco-2 cells. γC and its derived peptides (obtained via simulated gastrointestinal digestion) did not influence the cell viability at incubation times up to 24 h. The incubation of cells with native or digested γC caused no detectable inflammation processes mediated by Nuclear Factor kappa B (NFκB). We checked if treatment with γC or its derived peptides can elicit the expression of two peptide transporters (Pept-1 and Htp-1) by using an RT-qPCR approach. Native γC caused the halving of Pept-1 expression compared to untreated cells, but this effect disappeared when γC was digested. Either native γC or γC peptides reduced the expression levels of Hpt-1. Finally, this work also sheds light on the possible structural modifications of γC that may occur in the gastrointestinal tract, using an in vitro simulated dispersed system with polystyrene nanoparticles (NPs).

## 1. Introduction

γ-Conglutin (γC) from *Lupinus albus*, a legume plant widely used in human nutrition, accounts for 3%–4% of total seed proteins and is a tetrameric glycoprotein [1]. Each monomer is formed by two polypeptide chains of about 30 and 17 kDa, linked by a disulphide bond derived from a single precursor. The larger subunit is glycosylated. According to the deduced amino acid sequence (UniProtKB/TrEMBL accession number: Q9FSH9 LUPAL), its theoretical molecular weight is 45,446 Da and its pI is 8.24 [2].

γC has created remarkable pharmacological and/or nutraceutical interest. It was proven to significantly decrease glycaemia in animals and humans when orally administered [3,4,5] and to modify the gene expression of enzymes involved in hepatic glucose production [6,7]. The glucose-lowering effect of the protein was comparable to that obtained with metformin, the most commonly prescribed drug to treat patients suffering from prediabetes. The applicative repercussions of these findings are relevant, since lupin seeds are a common component of the Mediterranean diet [8], and thus γC might complement pharmacotherapy in the management of glucose intolerance [9].

Experiments using mouse myoblasts demonstrated that γC causes the activation of the same intracellular kinases of the insulin signaling cascade [10], suggesting a possible therapeutic indication for γC as an insulin-mimetic agent. γC was found accumulated inside the cytosol of the human model cells, such as Caco-2 monolayers and cultivated HepG2 [11,12]. The protein was also detected inside intestinal everted sacs [11]. In all these cases, the protein was found in an intact form. These findings indicate that γC may be translocated across the intestinal barrier.

It has been recently shown that γC has a high affinity with model phospholipid membranes [13]. This suggests that the protein may also interact with other hydrophobic systems. These interactions can entail the conformational remodeling of the protein structure with possible repercussions on its functionality. An in vitro simulation of in vivo conditions is usually achieved by reproducing the typical molecular crowding occurring in a biological system, depending on whether the environment is a cell or a food system. In such a complex system where proteins and peptides often exert their functions when inserted into macromolecular structures (such as protein complexes or membranes), or in food systems (when associated with dispersion phases), protein unfolding and misfolding is quite usual, whether incidental or induced. The balance between the loss and the gain of functions plays a crucial role in defining the protein’s molecular properties. The list of these properties is quite large, ranging from the in vivo proteotoxicity of misfolded proteins, to immunoreactivity, to altered techno-functional properties [14,15,16,17]. The stable adhesion of proteins to hydrophobic structures, such as nanoparticles (NPs) or nanoemulsions, is a way to mimic these complex systems [18,19]. NPs lead to conformational changes in proteins, permitting an exposure to new epitopes and, in general, a change in avidity capacity. Not only does the NP/protein stability vary according to the type of biological fluid in which they stay, but the protein concentration around the nanobeads also affects the binding reaction [20,21].

The present work continues past investigations on the cellular mechanisms underlying in vitro effects, focusing on the possible metabolic effects of the protein using cultivated Caco-2 cells, and shedding light on the possible structural modifications of γC that may occur when the protein reaches the gastrointestinal tract, adopting an in vitro simulated dispersed system using polystyrene NPs.

## 2. Materials and Methods

Reagents were from Sigma-Aldrich (Milan, Italy), if not otherwise specified. Polystyrene nanoparticles (diameter 200 nm, 2.5% (*w/v*) suspension) were from Kisker Biotech GmbH and Co. (Steinfurt, Germany).

### 2.1. Cell Cultures

Caco-2 cells were grown in 75 cm^2^ flasks at 37 °C in a humidified atmosphere of 6% CO_2_ in the air, using Dulbecco’s Modified Eagle’s Medium (DMEM) containing 10% fetal bovine serum (previously inactivated at 56 °C for 30 min), 2 mM L-glutamine, 100 U mL^−1^ penicillin, and 0.1 mg mL^−1^ streptomycin.

The day before transfection, cells were seeded in 24-well plates at a density of 2 × 10^5^ cells cm^−2^. Cells were transiently transfected with pNiFty2-Luc plasmids (InvivoGen, Rho, Italy) using the StoS transfection kit (GeneSpin, Milan, Italy), following the supplier’s protocol. After transfection, the cells were incubated in a complete DMEM medium in the presence of 80 µg mL^−1^ zeocin (InvivoGen) for 24 h at 37 °C in a humidified atmosphere of 6% CO_2_ in the air. Transfected Caco-2 cells were then incubated in 0.2 mL of fresh DMEM containing γC or NPs or a γC/NP complex at the indicated amounts. Interleukin 1β (IL1β) was used as a positive effector control at a final concentration of 10 ng mL^−1^. After 4 h, the multi-well plates were chilled for 15 min on ice, and then the cells were scraped from the bottom of the wells. The complete contents of each well were transferred into 1.5 mL test tubes placed on ice and sonicated three times for ten seconds using a Soniprep 150 Ultrasonic Disintegrator (MSE, Fisher Scientific, Loughborough, UK). After centrifugation to remove cell debris, a 0.1 mL aliquot of each supernatant was transferred into a 96-well microtiter plate well, and ATP and d-luciferin were added (final concentrations: 1.20 mM and 12 µM, respectively). The emitted luminescence was monitored every 120 s using a Victor3 Multilabel Counter (PerkinElmer, Waltham, MA, USA) at 560 nm. Each sample was analyzed in triplicate, using two biological replicas.

### 2.2. Cell Vitality Assays

The viable cell number was determined using a Cell Growth Determination Kit (Merck, cat. CGD1) on a 96-well multiplate according to the manufacturer’s instructions. This method is based on a (3-(4,5-dimethylthiazol-2-yl)-2,5-diphenyltetrazolium bromide (MTT) reduction, operating via mitochondrial dehydrogenase to form insoluble formazan, which was then spectrophotometrically determined at 570 nm upon solubilization with acidified isopropanol [22]. Each sample was analyzed in triplicate using two biological replicas.

For direct counts, cells were collected after peptide or protein treatment from multiplate wells using 0.4 mL cm^−2^ of trypsin/EDTA solution (Sigma-Aldrich, cat. T3924, Milano, Italy) and incubated at 37 °C for 5 min. Then, the suspension was place into a 1.5 mL tube, centrifuged at 15,000× *g* at RT (Eppenforf 5415R centrifuge equipped with an F24-15-11 fixed angle rotor), and the supernatant was discarded. Cells were resuspended in 1 mL of DMEM. Cell suspension aliquots were mixed with an equal volume of 0.4% Trypan Blue solution (*w/v*) (Sigma-Aldrich cat. 93595) in a test tube. Ten μL were placed on a TC20 Automated Cell Counter (Bio-Rad, Hercules, CA, USA) for automatic counting.

### 2.3. Gene Expression Relative Quantification

Caco-2 cells plated in a 24 well-plate, were raised to confluence and then treated for 3 h with 0.5 mg/mL of different protein samples (digested and not-digested). After incubation, the cells were lysed to extract the mRNA by using an Aurum total RNA kit, according to the manufacturer’s protocol (Bio-Rad, Hercules, CA, USA). The mRNA was retro-transcripted using iScript Reverse Transcription Supermix (Bio-Rad). Each cDNA (2 μL from the previous step) was amplified and quantified via real time PCR. Reactions were established in a final volume of 20 μL using iQ SYBR Green Supermix (Bio-Rad), with each primer added to a final concentration of 0.25 μM. The primer was designed by using the full sequences of pept-1 (5′-caaattaagatggttacgaggg-3′ and 5′-gaagatcgggaccatgatcacg-3), htp-1 (5′-acagactttgaggagagggcg-3′ and 5′-cagaagggtggtcagcagtatacc-3′), and the housekeeping actin as normalizer genes (5′-gcaccacaccttctacaatgag-3′ and 5′-tcacgcacgatttcccgctc-3′) [23,24,25]. The cycling conditions were as follows: 1 cycle at 95 °C for 5 min; 45 cycles of 95 °C for 20 s, 55 °C for 20 s, and 68 °C for 20 s. A no-template control with no genetic material and no retro-trascripted RNA was included to exclude contaminations or nonspecific reactions. Relative quantifications were calculated according to the Livak method [26], setting the gene expression levels of the untreated cells to 1.

### 2.4. Preparation of γC Samples and Interaction with Nanoparticles

γC was prepared to homogeneity as previously described [9], lyophilized, and stored at 4 °C in a desiccator. Before use, the protein was dissolved to a concentration of about 2 mg mL^−1^ in a sodium phosphate buffer of 50 mM, pH 7.3, or in sodium acetate buffer of 50 mM, pH 4.5, depending on the experimental conditions necessary for subsequent experimentation. The solution was centrifuged for 10 min at 15,000× *g* and precisely quantified spectrophotometrically at 280 nm, according to [27]. Sample tests were prepared using a γC final concentration of 0.5 mg mL^−1^ and increasing amounts of polystyrene nanoparticles (0.25, 0.50, 0.75, 1.00, and 1.50 mg) in the appropriate buffer. A control without the addition of nanoparticles was also prepared. Binding between γC and NPs was fostered by incubation of the samples at 25 °C at different times, with continuous gentle stirring. Then, the samples were centrifuged at 15,000× *g* for 30 min. Supernatants and precipitates were separated and analyzed, as described below.

### 2.5. SDS-PAGE

SDS-PAGE [28] was carried out as described on 12.5% polyacrylamide gels using a mini-Protean III cell (Bio-Rad, Hercules, CA, USA). Runs were carried out at a constant 16 mA for each gel. Polypeptides were visualized by Coomassie Brilliant Blue staining. The relative molecular weight of the polypeptides was determined by comparison with the standard proteins (GE-Healthcare, Milan, Italy) containing phosphorylase b (94 kDa), bovine serum albumin (67 kDa), egg albumin (45 kDa), carbonic anhydrase (29 kDa), trypsin inhibitor (20 kDa), and lysozyme (14 kDa).

Supernatants were analyzed as such, whereas pelleted NPs were resuspended at their initial volume with the appropriate buffer. Samples were mixed with an equal volume of the sample buffer (0.25 M Tris-HCl, pH 6.8, 7.5% glycerol, 2% SDS, and 2% β-mercaptoethanol) and heated at 100 °C for 5 min prior to being loaded onto the gel.

### 2.6. Proteolytic Digestions

Simulated gastrointestinal digestion was carried out by adapting a procedure previously described [29], focusing the action only on the proteolytic phase. Freeze-dried γC was resuspended (1 mg mL^−1^) in sterile water adjusted to pH 2 with 1 M HCl. Pepsin (Sigma-Aldrich, cat. P7012) was dissolved (0.5 mg/mL) in 10 mM Tris-HCl buffer, pH 6.5, containing 150 mM NaCl and added to protein samples at a ratio of 1:100. The samples were incubated at 37 °C for 10 min under shaking. After that, the pH was adjusted to 8 with a 1 M NaOH solution, and porcine pancreatin was added to samples at a ratio of 1:100. The samples were shaken again at 37 °C for 10 min. At the end, the reaction was stopped with a protease inhibitor cocktail (Sigma-Aldrich, cat. P8340).

For experiment with NPs, γC (0.5 mg) and 30 μL of the NP suspension were incubated in one mL of Tris-HCl 50 mM buffer at pH 8.0, at 20 °C for 30 min, with occasional gentle stirring. Samples were then centrifuged for 30 min at 15,000× *g*, and the supernatants were separated from the precipitate. To 250 μL of supernatant, 40 μL of trypsin stock (0.05 mg/mL, prepared in 2 mM HCl/NaCl, pH 3.0) was added. Instead, the precipitate was resuspended in 250 μL of Tris-HCl 50 mM buffer, pH 8.0, and 10 μL of trypsin stock was added. The sample control was prepared in the same way, without the addition of NPs. 50 μL of trypsin stock was added to the 250 μL control solution. Sample were incubated at 20 °C for different times (0, 15, 30, 45, and 60 min). The trypsin reaction was stopped by adding an equal volume of an electrophoresis sample buffer and rapid heating at 100 °C for 10 min, before SDS-PAGE was performed.

### 2.7. Protein Densitometric Quantification

The amount of each polypeptide separated by SDS-PAGE was quantified after CBB staining by computer-assisted image densitometry. Band intensities were determined using the open source software ImageJ (ver. 150e, National Institutes of Health, Bethesda, MD, USA), available at https://imagej.net/ImageJ [30], and expressed as arbitrary units. All determinations were carried out in triplicate, and the results were given as the mean ± standard error.

### 2.8. In-Silico Predictions

Prediction of the peptides’ toxicity was carried out using the ToxinPred method [31], available on-line at http://crdd.osdd.net/raghava/toxinpred/, using the Uniprot KB: Q9FSH9 amino acid sequence of γC as a template.

### 2.9. Statistical Analysis

Data reported in the histograms are expressed as the means ± S.E. Data were analyzed by a t-test. *p* values < 0.05 were considered to be statistically significant. Data from RT-qPCR were analyzed by using the CFX Maestro software (Bio-Rad, Hercules, CA, USA).

## 3. Results and Discussion

As stated before, many aspects of the possible bioactivities of γC on model human cells have yet to be further elucidated. We focused on the effects on the Caco-2 cell, since previous works on the subject have been carried out using this cell model [11]. Caco-2 is a cell line derived from human colon adenocarcinoma used as a common model of the intestinal barrier for in vitro toxicology studies and to test the biological activities of food-derived molecules [32].

### 3.1. Cytotoxicity Assessments

**Figure 1:**
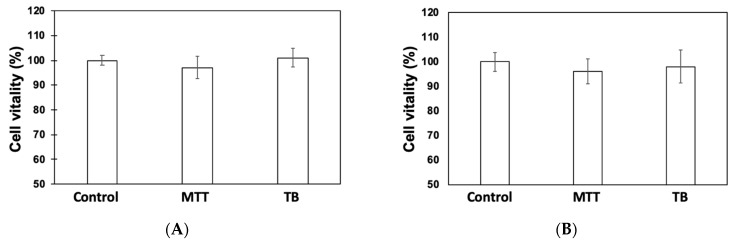
Assessment of Caco-2 cell vitality following treatment with native γC (**A**) and with γC-derived peptides (**B**) using the 3-[4–dimethylthiazol-2-yl]-2,5-diphenyltetrazolium bromide (MTT) method and direct cell counting (Tryphan Blue (TB)). Data are expressed as a percentage of the control sample.

Previous works have shown, by confocal microscopy and TEM, that γC aggregates stack onto the cell membranes and are found inside the cytoplasm in an intact form [12]. This may be the prerequisite for exerting a biological function, but possible parallel negative effects cannot be excluded. We verified whether any cytotoxicity of γC towards Caco-2 cells could became evident at incubation times up to 24 h, since, in previous works, incubations were generally limited to 3 or 6 h of protein/cell contact. Cell loss and metabolic activities were assessed using Tryphan Blue (TB) and 3-[4–dimethylthiazol-2-yl]-2,5- diphenyltetrazolium bromide (MTT). The results are shown in Figure 1. No significant losses were visible, indicating that γC itself does not influence the cell’s viability at prolonged incubation times (Figure 1A). Moreover, it was clearly established that ingested food proteins may release toxic peptides following the digestion process [33,34]. In-silico prediction indicated that some regions of γC, located on the large subunit at positions around 180–196, can generate potentially toxic peptides (Supplementary Materials, Figure S1). However, our experimental results seem to exclude this evidence. Indeed, when Caco-2 cells were treated with the peptides obtained by the in vitro-simulated gastro-intestinal digestion of γC, no negative effects were observed (Figure 1B).

### 3.2. γC effects on Inflammatory Pathways

**Figure 2:**
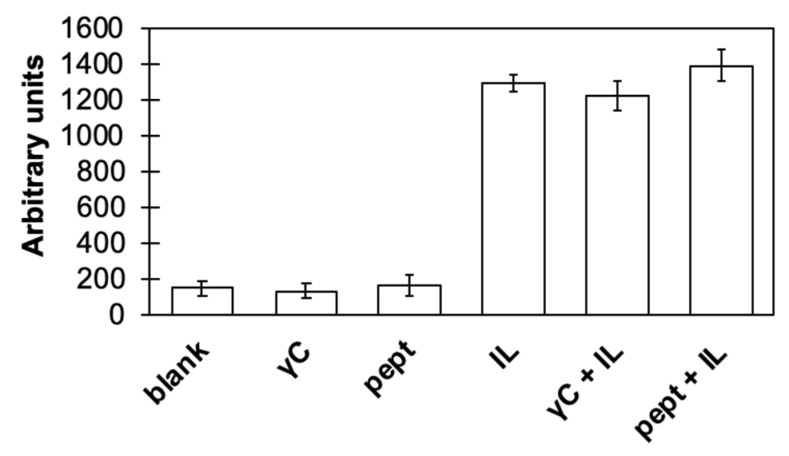
Effects on the Caco-2 transfected cell nuclear factor kappa B (NFκB) pathway of *γC* and of its peptides derived from simulated gastrointestinal digestion (pept). Experimental details are given in the text. Blanks are Caco-2 cells incubated alone (negative control), whereas IL are cells incubated with Interleukin 1β (IL1β) (positive control). γC and pept have been also been incubated in the presence of IL1β (+IL).

The possible effects of γC on inflammation cell pathways has been studied by using transfected Caco-2 cell expressing markers of the immune responses triggered by interleukin 1 beta (IL1β) through the activation of the nuclear factor kappa B (NFκB) pathway [35]. NF-κB is implicated in many physiological processes, such as the innate and adaptive immune response, cell death, and inflammation. It also activates cytokine transcription. The results reported in Figure 2 show that the incubation of Caco-2 cells with native or in-vitro digested γC caused no detectable inflammation processes. This indicates that the protein or its proteolytic breakdown peptides are not able to mime IL1β stimulation (that is, they do not activate the NFkB pathway). On the other hand, if the cells were incubated with IL1β, the presence of either γC or peptides did not influence the inflammatory process.

### 3.3. Effects on the Expression of Intestinal Peptide Transporters

**Figure 3:**
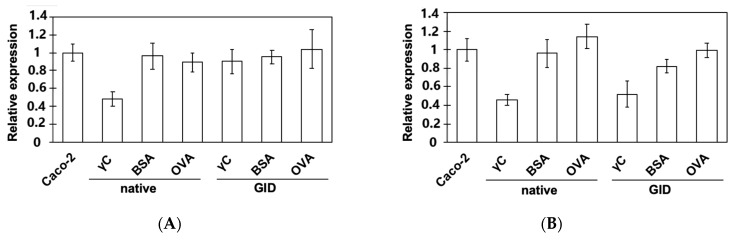
The modulation of intestinal peptide transporter gene expression in the Caco-2 cells of γC, determined by RT-qPCR. (**A**) Expression modulation of the Pept-1 transporter. (**B**) Expression modulation of the Hpt-1 transporter. Chicken ovalbumin (OVA) and bovine serum albumin (BSA) were also tested. Proteins have been tested in native conditions and after simulated gastrointestinal digestion (GID). Caco-2 are untreated cells.

The addition of a protein to the incubation medium could have repercussions on the metabolism of the cells. We checked if treatment with γC or its derived peptides can elicit, on Caco-2 cells, the expression of two main peptide transporters (Pept-1 and Htp-1) by using an RT-qPCR approach. The results are shown in Figure 3. Cells were also treated with chicken ovalbumin (OVA) and bovine serum albumin (BSA), at the same concentration (0.5 mg mL^−1^), for comparison and to exclude generic aspecific artifacts. It has been well esthablished that incresing the protein concentration influences the transcription and activity of these transporters [36,37,38]. A comparison between intact and hydrolyzed proteins was also made using the same final protein concentration (0.5 mg mL^−1^).

Pept-1 is an oligopeptide transporter located on the cellular membrane of Caco-2 and other human intestinal cells [36], whose expression is increased by several kinds of molecules but not insulin. Rather, this hormone stimulates peptide transport by increasing the amount of Pept-1 on the membranes from a preformed cytoplasmatic pool [39]. γC shares common effects with insulin, as it is able to regulate gene expression, energy metabolism, and protein synthesis through the modulation of the same insulin signalling pathways [10]. Interestingly, the presence of γC in the medium caused the halving of the transporter expression recorded in untreated cells (Figure 3A). On the other hand, this effect desappears when γC is digested, with expression levels similar to those of the control cells. Either native or digested OVA and BSA have no significant consequences on the expression of Pept-1 under the adopted experimal conditions. The fact that native γC shows the downregulation of Pept-1 is surprising and intriguing, since it is not one of the expected effects of any molecule described, so far, as an insulin analog [4,10,39]. Peptides that are generated by the simulated gastrointestinal digestion of the three proteins are not able to modulate Pept-1 expression. However, the possible translocation of the preformed Pept-1 to the cell’s apical membranes cannot be excluded [38]. However, in light of these results, consequences due to cell growth conditions may be ruled out [40].

Hpt-1, the second main peptide transporter, is expressed on apical membranes in Caco-2 cells and is also known as cadherin CDH17 [27]. Aberrant expressions of CDH17 have been associated with cancer development and progression [41]. Our results (Figure 3B) indicate that treatment with either native γC or γC peptides reduced the expression level of Hpt-1, and, meanwhile, the intact OVA and BSA have no influence. Digested BSA peptides seem to exert a slight downregulation of this transporter. Whether these data are relevant for tumor prevention or progression issues has yet to be established and needs future, more, focused investigations.

### 3.4. γC molecular Features and Biological Effects Following Adsorbtion to Polystyrene NPs

**Figure 4:**
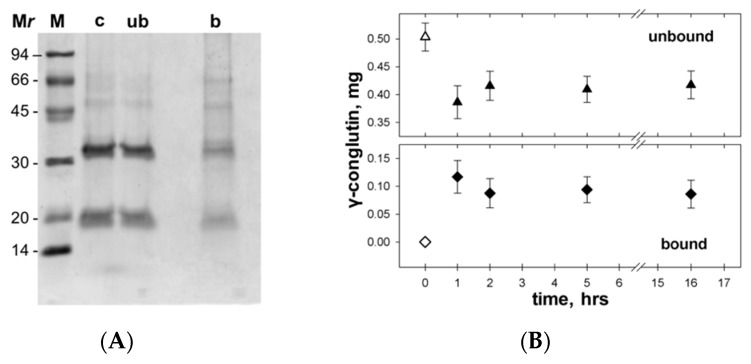
Adsorption of γC to polystyrene nanoparticles (NPs). Experimental details are given in the text. (**A**) SDS-PAGE of γC used for the experiment (c), unbound (ub), and bound (b) to NPs. The M*r*s of the markers (M) are given in kDa; (**B**) quantification of γC remained unbound (closed triangles) or bound (closed diamonds) to the NPs during incubation. Open symbols indicate the initial amounts of protein.

As the first trial, we tested the binding properties of γC to NPs. Total of 30 µL (0.75 mg) of NP suspension was added to 0.5 mg of protein and incubated, at pH 7.3, at room temperature for 45 min. After centrifugation, both the supernatant and the precipitated NPs were analyzed. SDS-PAGE (Figure 4A) showed that the protein effectively interacted with NPs. The adsorption was quite fast and stable over time. By incubating the samples at different time intervals (1, 2, 5 h, and overnight), the amount of bound γC (i.e., the decrease of soluble unbound γC in the supernatant, detected by spectrophotometric reading at 280 nm) did not vary significantly (Figure 4B).

**Figure 5:**
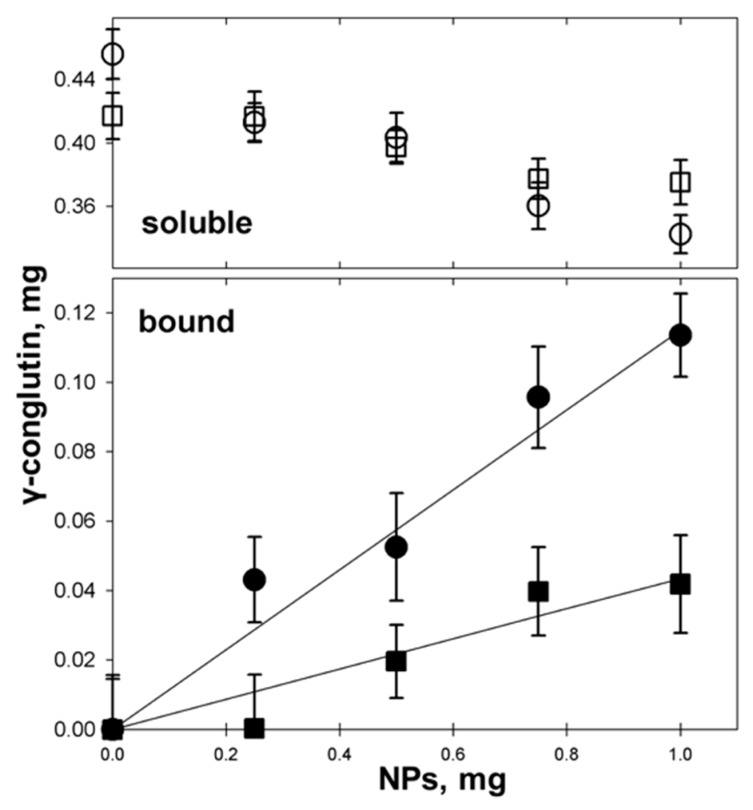
Absorption of γC to polystyrene NPs at pH 7.3 (dots) and pH 4.5 (squares). Upper panel: soluble γC after incubation with NPs (open symbols); lower panel: NP-bound γC (closed symbols).

To quantify the observed binding, increasing amounts of nanoparticles (0.25, 0.50, 0.75, 1.00, and 1.50 mg) were mixed with a fixed amount of γC (0.5 mg) in one mL of an appropriate buffer and incubated for one hour, as described above. After centrifugation, the bound protein was calculated by its difference from the amount of unbound protein soluble in the supernatant (Figure 5). γC is adsorbed onto the hydrophobic surface of NPs according to a linear correlation.

At pH 7.3, where the protein is tetrameric, the adsorption ratio is 115 ± 10 µg of protein per mg of NPs. If γC is instead adsorbed to NPs in an acetate buffer (pH 4.5), where it is in a monomeric form, the binding ratio reduced to 43 ± 6 µg of protein per mg of NPs, which is about half of that bound at pH 7.3. Hypothesizing that, at both pHs, the NP surface is saturated by proteins, these results suggest that at acidic pHs, γC is adsorbed as a monolayer onto hydrophobic NPs. Conversely at a neutral pH, taking advantage of its multimeric form, a bilayer structure is likely assembled. On the other hand, on a stoichiometric basis, more protein is bound to NPs in an acidic pH.

**Figure 6:**
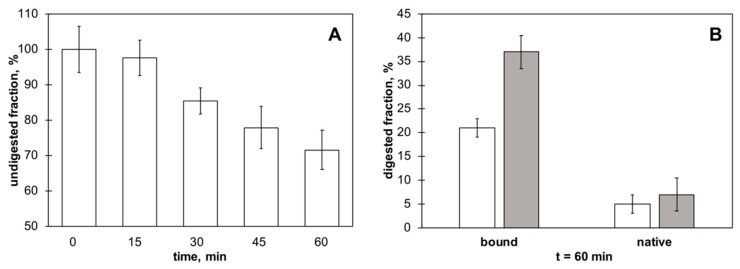
The adsorbed γC’s sensitivity to trypsin and its constituent subunits. (**A**) A relative amount of γC remained bound to the NPs over time; (**B**) the digested fraction of 30 kDa (white bars) and of 17 kDa (gray bars) subunits of γC bound to NPs or alone after 60 min of incubation with trypsin.

Different methodologies can be adopted to verify the possible conformational changes of γC following its adsorption to polystyrene nanoparticles. γC shows a rather unusual resistance to proteases, both during seed germination processes and in in vitro tests [42]. At pH values below 4.25, a relevant rearrangement of the protein conformation occurs [1], resulting in a marked susceptibility of γC to enzymatic hydrolysis, while it is completely resistant to various proteolytic enzymes and protease mixtures at higher pH values. Thus, trypsin was used to assess the possible conformational changes of γC following its adsorption to NPs. This experimental approach has been successfully applied in previous works testing the structural variations of γC [43]. After adsorption, γC/NPs were incubated with trypsin for up to 24 h (Figure 6).

The amount of protein bound to NPs decreased during the treatment (Figure 6A), indicating that trypsin can act on immobilized γC. As expected, trypsin did not digest native protein in the solution without NPs (Supplementary Materials, Figure S2). No breakdown products are visible as discrete bands on the SDS-PAGE (Supplementary Materials, Figure S2B), at least for the NP-bound protein or the protein alone after 60 min of incubation with trypsin. The adsorbed γC, being more digestible than the native protein, likely takes a less compact tertiary structure than the protein in the solution (Figure 3B). The 17 kDa subunit of γC is more accessible to trypsin than the 30 kDa subunit (Figure 3B). Overall, these results allow us to depict a possible sequence of events. Binding to NPs leads to conformational changes of the protein, giving access to regions of the polypeptide chain containing buried K and R residues that are otherwise inaccessible to the protease mainly located on the small subunit and opening the way to an advanced level of enzymatic digestion. Whether digestion modifies the capacity of γC to interact with the hydrophobic surface of NPs remains to be directly established, even if no significative differences were recorded in the amounts of soluble γC released from γC/NPs during the time course of the enzymatic treatment.

**Figure 7:**
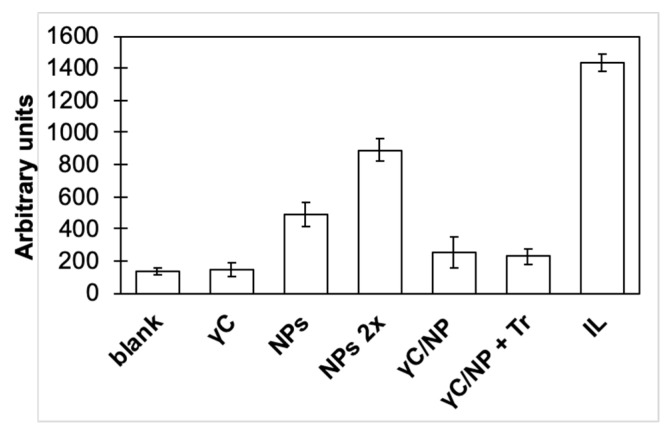
Effects on the Caco-2 transfected cell NFkB pathway of the γC, NP, and γC/NPs complex. γC/NPs+Tr was the complex treated with trypsin for 60 min, as shown in Figure 6. See the text for experimental details. Blank areas are the Caco-2 cells incubates alone (negative control), whereas IL is the cells incubated with IL1β (positive control).

The possible variations of the biological properties of γC have been assessed by checking the possible inflammatory/anti-inflammatory properties of the transfetted Caco-2 cells variations. The results are shown in Figure 7.

The contact of NPs alone with the Caco-2 cells caused a triggering of the inflammatory cascade. This pro-inflammatory effect appears to be dose dependent; by doubling the quantity of NPs, the inflammation increased twice. NPs made of different materials showed a similar effect in their fibroblasts, macrophages, and bronchiolar epithelial cells, as previously described [44]. Interestingly, the absorption of γC to polystyrene NPs masked the inflammatory effects of NPs. This effect remains even if the protein has been affected by the action of trypsin. Although naked and conjugated NPs share very close dimensions (the thickness of the protein layer is expected to be negligible compared to the diameter of the NPs), naked NPs present a very hydrophobic surface that directly interacts with Caco-2 cells. On the other hand, in conjugated NPs, the protein coating acts as a surfactant, hiding the NPs’ surface and mediating the NPs’ interactions with cells.

## 4. Conclusions

In the last few years, the research of bioactive proteins and peptides derived from foods has increased. Our studies on γC fit into this workflow. The new findings reported in the present work are relevant to delineating a deeper understanding of the metabolic fate of orally administered γC and addressing its applicative exploitation. Of course, our results open the way to further studies aimed at ascertaining the precise molecular determinants underlying the hypoglycemic activity of γC. Moreover, this study adds one piece to the puzzle of the possible uses of nanomaterials in food systems. Hydrophobic polystyrene NPs have been proven to be pro-inflammatory for Caco-2 cells. γC-coated NPs completely lose this negative trait, suggesting that the nanometric dimensions, per se, are not responsible for NFkB pathway stimulation and underscore the relevance of the NPs’ nature.

From a methodological point of view, it must be underscored that working with NPs does not allow one to apply the usual spectroscopic techniques (such as spectroscopy, spectrofluorimetry, and spectropolarimetry) to characterize the protein’s structural features. Consequently, all this information must be acquired by indirect approaches. For example, in this work, the amount of NP-bound protein was quantified by its difference to the protein fraction that does not bind to the NPs. Again, the protein’s susceptibility to hydrolysis with trypsin was employed as a structural probing tool. Taken together, our results show that the spontaneous adsorption of γC onto a hydrophobic surface goes alongside a rearrangement of the protein’s structure, which changes the reactivity of the protein. The resulting NP-protein corona, with respect to the native protein, has distinctive features that affect degradation and, more important to our studies, inflammation [45].

## Supplementary Materials

Supplementary materials can be found at https://www.mdpi.com/2218-273X/9/10/606/s1. Figure S1: Prediction of peptides toxicity calculated by using the ToxinPred method [31], available on-line at http://crdd.osdd.net/raghava/toxinpred/, using Uniprot KB:Q9FSH9 amino acid sequence of γC as template, Figure S2: Panel A: native γC incubated with trypsin for different times. Panel B: Incubation with trypsin effects on γC adsorbed to NPs.

## Abbreviations

NPs	Nanoparticles
γC	γ-Conglutin
MTT	3-[4–dimethylthiazol-2-yl]-2,5-diphenyltetrazolium bromide
NFkB	Nuclear Factor kappa B
TB	Tryphan Blue
IL1β	Interleukin 1β
