# Supplementary material for: Effects on the Caco-2 Cells of a Hypoglycemic Protein from Lupin Seeds in a Solution and Adsorbed on Polystyrene Nanoparticles to Mimic a Complex Food Matrix
Source: Biomolecules. 2019 Oct 14;9(10):606. doi: 10.3390/biom9100606 (PMC6843813; doi:10.3390/biom9100606)
Supplement: Supplementary file 1 [file biomolecules-09-00606-s001.pdf]

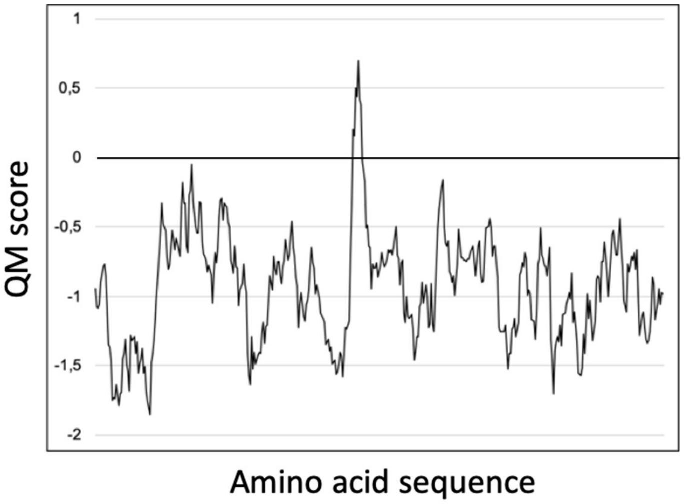

Supplementary Figure S1: Prediction of peptides toxicity calculated by using the ToxinPred method [31], available on-line at http://crdd.osdd.net/raghava/toxinpred/, using Uniprot KB:Q9FSH9 amino acid sequence of γC as template.

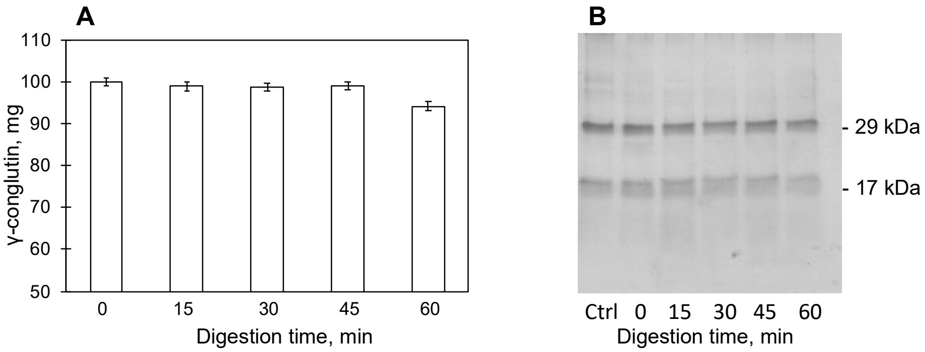

Supplementary Figure S2. Panel A: native γC incubated with trypsin for different times. **Panel B:** Incubation with trypsin effects on γC adsorbed to NPs. Ctrl stands for native γC incubated with trypsin after 60 min.
